# Canagliflozin-Induced Diabetic Ketoacidosis: Case Report and Review of the Literature

**DOI:** 10.1177/2324709616663231

**Published:** 2016-08-29

**Authors:** Jessica Turner, Tahmina Begum, Roger D. Smalligan

**Affiliations:** 1 Texas Tech University Health Sciences Center, Amarillo, TX, USA

**Keywords:** diabetic ketoacidosis, canagliflozin, SGLT-2 inhibitors

## Abstract

**Introduction:** Sodium-glucose co-transporter 2 (SGLT-2) inhibitors are relatively new antihyperglycemic agents that lower renal glucose reabsorption. They are used as adjunctive therapy to standard diabetes treatment. **Case Report:** We present the case of a 62-year-old woman with a past medical history of type 2 diabetes mellitus and sudden-onset diabetic ketoacidosis (DKA). Use of canagliflozin, a SGLT-2 inhibitor, was determined to be the cause of the DKA. The patient ultimately recovered after 5 days in the intensive care unit. She was changed to long- and short-acting insulins and instructed to avoid canagliflozin. **Conclusion:** Although SGLT-2 inhibitors are effective at lowering a patient’s hemoglobin A1C, physicians must be aware of the rare but dangerous potential adverse effect of inducing DKA. This article reports an illustrative case and presents a review of the literature.

## Introduction

**Figure 1.**
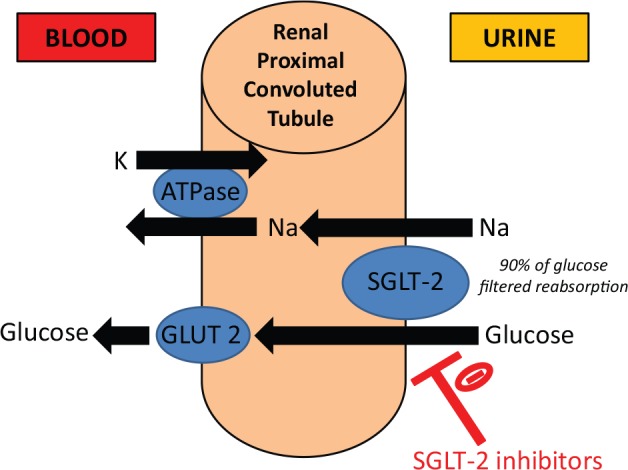
Mechanism **of action of canagliflozin on SGLT-2 in the renal proximal tubule.**

As of 2012, 29 million people in the United States suffered from diabetes mellitus.^1^ Sodium-glucose co-transporter 2 (SGLT-2) inhibitors are a new class of antihyperglycemic agents used as adjunctive therapy to standard treatment regimens. SGLT-2 inhibitors are sold as both single-ingredient products and also in combination with metformin and other diabetic medications. These drugs lower renal glucose reabsorption and increase urinary glucose excretion^2^ (Figure 1).

Common side effects of SGLT-2 inhibitors include genital yeast infections, urinary tract infections, and hypotension. However, in May 2015, the Food and Drug Administration (FDA) issued a drug safety communication which reported that these medications may lead to diabetic ketoacidosis (DKA).^3^ We present the case of a previously stable patient with type 2 diabetes mellitus who was treated with canagliflozin and developed severe DKA.

## Case Report

A 62-year-old woman with type 2 diabetes mellitus, hypertension, gastroesophageal reflux disease, and depression presented with 4 days of nausea, vomiting, and generalized weakness. Her symptoms became progressively worse such that by the day of admission she had decreased appetite, polydipsia, polyuria, and could not walk. The patient denied fever, chills, abdominal pain, diarrhea, or sick contacts. Home medications were atorvastatin, metformin, sucralfate, pioglitazone, canagliflozin, exenatide, omeprazole, fluoxetine, ranitidine, lisinopril, and alprazolam.

On physical examination, the patient’s vital signs included a temperature of 38.3°C, blood pressure 134/61, heart rate 107, respiratory rate 24, and oxygen saturation of 100% on 2 liters nasal cannula oxygen. The patient appeared ill and distressed. She had dry mucous membranes, clear lung sounds bilaterally, and her heart was regular without murmurs, gallops, or rubs. Her abdomen was soft and nontender with present bowel sounds. Extremities showed no edema, and she had no focal neurological findings. Laboratory revealed a metabolic acidosis with a pH of 7.08 and anion gap >17. Chemistry panel indicated sodium 134 mEq/L, potassium 5.2 mEq/L, chloride 112 mEq/L, CO_2_ <5 mEq/L, blood urea nitrogen 22 mg/dL, and creatinine 1.3 mg/dL. Blood glucose was 213 mg/dL, and urinalysis revealed glucose 2+ and ketones 3+. Serum ketones were present at 1:8 dilution, with a lactic acid of 0.8 mmol/L. The patient’s hemoglobin A1C (HbA1c) was 11.1.

The patient was admitted to the intensive care unit for severe metabolic acidosis secondary to DKA. Aggressive fluid resuscitation was undertaken and an insulin drip initiated. Within 6 hours, the anion gap metabolic acidosis improved. On further review of her medication history, it was discovered that canagliflozin had been started several months prior. Further study of that medication and its uncommon side effects led to the etiology of the DKA. The patient required 5 days of hospitalization for complete resolution of her symptoms. She was sent home with a regimen of long- and short-acting insulins, as well as instructions to avoid canagliflozin. Her endocrinologist was advised of this adverse reaction.

## Discussion

DKA is a rare but serious adverse event seen increasingly with canagliflozin and other SGLT-2 inhibitors.^4-10^ The FDA Adverse Event Reporting System (FAERS) database identified 73 cases of DKA from March 2013 through May 2015 for patients using this class of medications. Though 73 cases have been officially reported to the FAERS database, it is likely that there are additional cases of patients that required hospitalization for DKA. In fact, on notifying our patient’s endocrinology office, the nurse commented that they had seen 2 other similar cases recently from their office practice. At least 15 of the reported cases were type 1 diabetics, suggesting off-label use of canagliflozin. Canagliflozin is only FDA approved for type 2 diabetes treatment. A revised drug label reporting the risk of new DKA with the use of these medications has since been issued by the FDA.^11^ Patients should be advised to look closely for any signs of ketoacidosis including shortness of breath, nausea, vomiting, abdominal pain, altered mental status, and fatigue. If signs of DKA are present, patients should be advised to stop taking their SGLT-2 inhibitor and seek immediate medical attention. The median time of DKA onset was 43 days, though a range of 1 day to 1 year has been reported.^11^ All 73 reported cases of canagliflozin-induced DKA required hospitalization.

It is still unknown why there appears to be DKA development associated with canagliflozin use. Prior research has found that SGLT-2 inhibitors are associated with an increase in plasma glucagon levels.^12^ The mechanism behind this is unknown. Although somewhat complex, one hypothesis regarding the occurrence of DKA in this setting is that some patients may be misdiagnosed as type 2 diabetics when they actually have an antibody-mediated ketoacidosis prone type of diabetes giving them low beta cell reserves. This would lead to insufficient insulin levels when a patient is stressed by an acute illness. In the setting of SGLT-2-induced increased glucagon levels, this would permit ongoing hepatic ketogenesis and peripheral lipolysis, leading to the DKA.^13^

Typically DKA is associated with hyperglycemia above 250 mg/dL; however, for many patients in the reported cases and in ours, the blood glucose levels were lower, with a mean of 211 mg/dL.^11^ This euglycemic DKA (euDKA) may be underrecognized and result in delayed treatment with the necessary aggressive intravenous hydration and insulin.^13^ It is always important to rule out other potential causes of DKA including major illness, dehydration, and inadequate insulin intake as was done in our case. The dosage of SGLT-2 inhibitors does not appear to correlate with development of new DKA.^11^

Since the release of the FDA drug safety communication, the safety of canagliflozin and the risk of DKA development have been studied using large databases of patients treated. Erondu et al^14^ analyzed a database of 17,596 patients from 15 trials assessing the adverse effects of canagliflozin therapy. The incidence of DKA and related events (ketoacidosis, metabolic acidosis, and acidosis) was 0.07%, corresponding to 12 events out of the 17,596 patients. Of these 12 patients, 4 had received 100 mg canagliflozin, 6 had received 300 mg canagliflozin, and only 2 patients were in the comparator arm of the study. However, of the 10 patients taking canagliflozin who developed DKA or a related event, 6 patients were later discovered to have autoimmune diabetes such as latent autoimmune diabetes, type 1 diabetes, or a positive GAD65 antibody. The authors do acknowledge the possibility of underreporting of events and are fair to report that the study was funded by the manufacturer.

To date there is not an established phenotype to help clinicians accurately predict the risk of DKA events when using an SGLT-2 inhibitor.^14^ However, the risk of DKA is higher in the following groups: older white males and those with a lower body mass index, higher HbA1c level, lower glomerular filtration rate, and longer duration of diabetes. However, *P* values are not reported in the table listing these risk factors.^14^ Our patient was a 63-year-old female with a body mass index of 26.63, HbA1c 11.1%, estimated glomerular filtration rate 41.5 mL/min/1.73 m^2^, and an unknown duration of diabetes. Other than being female, all of the details mentioned for our patient fit the risks seen in other studies.

Though our patient was febrile and had episodes of vomiting, these are common symptoms of DKA; and testing was negative for a source of infection or other medical problems that could have led to the event. Specifically, she did not report abdominal pain, icterus, diarrhea, or sick contacts. Her urine culture, blood cultures, chest X-ray, troponins, liver function tests, and lipase were all unremarkable. Though other studies have linked alcohol usage with development of DKA, our patient denied alcohol intake and had a negative blood alcohol level and urine toxicology screen.^13^

## Conclusion

As canagliflozin is prescribed more and more by clinicians due to its proven ability to lower HbA1c levels, physicians must be aware of the potential, albeit uncommon, dangerous side effect of new DKA in a patient with type 2 diabetes, even months after initiating the drug. Further research and monitoring of adverse events is clearly needed to determine the frequency and severity of these reactions.^15^
